# Controlling the Self-Assembly of Biomolecules into Functional Nanomaterials through Internal Interactions and External Stimulations: A Review

**DOI:** 10.3390/nano9020285

**Published:** 2019-02-18

**Authors:** Li Wang, Coucong Gong, Xinzhu Yuan, Gang Wei

**Affiliations:** 1 Key Laboratory of Preparation and Application of Environmental Friendly Materials (Jilin Normal University), Ministry of Education, Changchun 130103, China; 15144435516@163.com; 2 Faculty of Production Engineering, University of Bremen, D-28359 Bremen, Germany; ccgong@uni-bremen.de

**Keywords:** self-assembly, biomolecules, nanostructures, interactions, external stimulations

## Abstract

Biomolecular self-assembly provides a facile way to synthesize functional nanomaterials. Due to the unique structure and functions of biomolecules, the created biological nanomaterials via biomolecular self-assembly have a wide range of applications, from materials science to biomedical engineering, tissue engineering, nanotechnology, and analytical science. In this review, we present recent advances in the synthesis of biological nanomaterials by controlling the biomolecular self-assembly from adjusting internal interactions and external stimulations. The self-assembly mechanisms of biomolecules (DNA, protein, peptide, virus, enzyme, metabolites, lipid, cholesterol, and others) related to various internal interactions, including hydrogen bonds, electrostatic interactions, hydrophobic interactions, π–π stacking, DNA base pairing, and ligand–receptor binding, are discussed by analyzing some recent studies. In addition, some strategies for promoting biomolecular self-assembly via external stimulations, such as adjusting the solution conditions (pH, temperature, ionic strength), adding organics, nanoparticles, or enzymes, and applying external light stimulation to the self-assembly systems, are demonstrated. We hope that this overview will be helpful for readers to understand the self-assembly mechanisms and strategies of biomolecules and to design and develop new biological nanostructures or nanomaterials for desired applications.

## 1. Introduction

Self-assembly is a simple but effective bottom-up technique for preparing functional nanomaterials with ordered structures and novel functions [1,2,3]. Besides nanoparticles (NPs) [4], polymers [5], and other inorganic nanoscale building blocks, many kind of biomolecules in nature, including DNA [6], proteins [7], peptides [8,9], viruses [10,11], enzymes [12,13], and others [14], have also exhibited great potential to form hierarchical nanomaterials by controllable self-assembly. Due to the unique molecular properties, adjustable functions, and ordered structures, the self-assembled biological nanomaterials have been widely utilized for applications in the fields of materials science, biomedical engineering, tissue engineering, biosensors, and nanotechnology [15,16,17,18,19,20].

In order to fabricate functional biological nanomaterials, one of the key challenges is how to control the self-assembly of biomolecules to form desired structures. Previous studies have indicated that this challenge could be solved through adjusting internal molecule–molecule/materials interactions (such as hydrogen bonding, electrostatic interaction, hydrophilic/hydrophobic interaction, and DNA/RNA hybridization [21,22,23,24]) or carrying out external stimulations (such as adjusting the pH, temperature, or ionic strength or adding organics and enzymes to the system [25,26,27,28]). To further improve the functions and applications of self-assembled biomolecular nanomaterials, some functional nanoscale building blocks, such as nanoparticles [29,30], carbon nanotubes [31], graphene [32,33], and polymers [34,35], could be introduced into the biomolecular self-assembly systems, where the potential biomolecule–materials (building blocks) interactions could guide the self-assembly of both biomolecules and the corresponding building blocks into hybrid nanomaterials. For instance, Yu et al. demonstrated the biomolecule-assisted self-assembly of CdS/MoS_2_/graphene hollow spheres for high-efficiency and low-cost photocatalysis [30]; Zou and co-workers reported the fabrication of novel nanodots by the self-assembly of peptide–porphyrin conjugates [35].

The self-assembly mechanisms of biomolecules to various nanostructures have been investigated widely, and some reviews on the design, synthesis, and applications of self-assembled biomolecular nanomaterials have been reported previously [36,37,38,39,40]. For example, Yang and co-workers provided an overview on the self-assembly of proteins to various supramolecular materials, in which the design strategies for self-assembling proteins were introduced and discussed in detail [39]. Willner et al. summarized the applications of biomolecule-based nanostructures and nanomaterials for sensing and the fabrication of nanodevices [40]. After studying these reports, we realized that it is still valuable for us to contribute a review on the self-assembly of biomolecules to functional nanomaterials from the viewpoints of internal interaction mechanisms, external stimulation, and designed functionalities.

In this review, we focus on the fabrication of biological nanomaterials by controlling the self-assembly of a biomolecule through internal biomolecular interactions and external stimulations. The main used/studied biomolecules, including proteins [7,41], peptides [42,43], amphiphiles [44], DNA [45], carbohydrates, metabolites [46,47,48], lipids, and cholesterol, for the self-assembly of various nanostructures are introduced and discussed in detail. In Section 2, some recent studies are analyzed and discussed to illustrate the self-assembly mechanisms of various biomolecules, in which the strategies for creating biological nanomaterials via internal interactions are presented. In Section 3, we introduce recent studies on adjusting biomolecular self-assembly via external stimulations. In Section 4, we provide a summary on the synthesis of biological nanomaterials based on various biomolecules. It is expected that this work will be helpful for readers to understand the self-assembly mechanisms of, and strategies for creating, functional nanomaterials, and to design and develop new biological nanostructures and nanomaterials for advanced applications in materials science, biomedical engineering, analytical science, energy, and environmental science.

## 2. Internal Interactions towards Biomolecular Self-Assembly

The mechanisms of biomolecules self-assembled into various nanostructures are complex. In this section, we demonstrate the self-assembly mechanisms of biomolecules from basic molecular interactions (such as hydrogen bonds and electrostatic, hydrophobic, and π–π interactions) as well as biomolecular-specific interactions (such as DNA base pairing, ligand–receptor/antigen–antibody binding, and biomolecule–polymer conjugates).

### 2.1. Basic Molecular Interactions

#### 2.1.1. Hydrogen Bonds

Hydrogen bonds, the interactions that occur between hydrogen atoms and a great number of electronegative atoms in biomolecules, play important roles in the formation of biological nanomaterials [49,50,51]. In the process of biomolecular self-assembly, the hydrogen bonds can promote the growth of biomolecules in one direction with a long-range order to form one-dimensional (1D) nanostructures. In addition, the hydrogen bonds between the hydrogen atoms of biomolecules and the electronegative atoms of a special material’s surface could enhance the interactions between biomolecules and materials for the formation of functional biomolecule-based hybrid nanomaterials.

**Figure 1:**
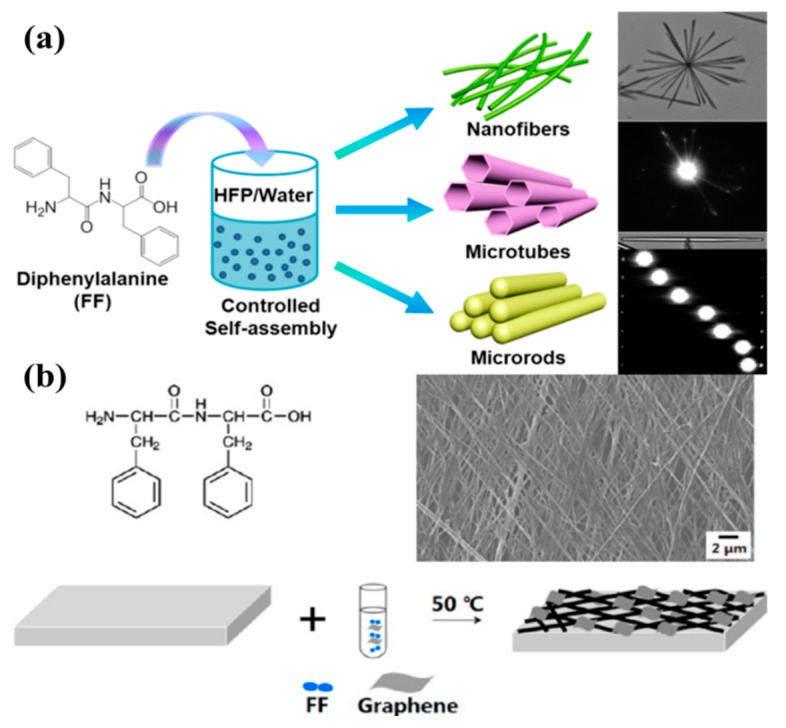
Hydrogen bonds promoted the self-assembly of biomolecules: (**a**) Hydrogen-bond-induced self-assembly of FF into nanofibers, microtubes, and microrods. Reprinted with permission from [21]. Copyright 2015 American Chemical Society. (**b**) The formation of microscale peptide nanowires (PNWs)–graphene array. Reprinted with permission from [52]. Copyright 2013 American Chemical Society.

Diphenylalanine (FF) is a popular dipeptide motif for self-assembly in water driven by hydrophobic interactions; however, other interactions also likely play a role. For instance, Li et al. demonstrated the formation of FF microrods by hydrogen-bond-based self-assembly [21]. The structural and property characterizations of the self-assembled microrods indicated that 1,1,3,3,6,6-Hexafluoro-2-propanol (HFP) formed stable intermolecular hydrogen bonds with an FF peptide, leading to the solvation of peptide molecules. When the peptide was dropped onto a silicon wafer, the evaporation of HFP promoted the self-assembly of FF to form nanofibers, microtubes, and microrods, as shown in Figure 1a. It can be found that FF molecules first self-assemble into nanotubes in the presence of water, and then grow into microrods through both hydrogen bonds and hydrophobic interactions between aromatic residues of the peptide. In another study, Yang and co-workers investigated the self-assembly of an FF peptide on a graphene surface and the formation of peptide nanowires (PNWs) [52]. Firstly, the peptide solution was diluted with a certain concentration of graphene dispersion solution and then dropped onto a clean substrate to dry in an oven at 50 ℃, as shown in Figure 1b. The self-assembly of the FF peptide with graphene in water was ascribed to both hydrogen bonds and π–π interactions. It can be concluded that graphene promoted the π–π conjugations between peptides and graphene, and the intermolecular/molecule–graphene hydrogen bonds mediated the formation of ordered PNW arrays on the graphene’s surface.

Besides peptides, proteins, enzymes, DNA, and viruses can also be utilized to form self-assembled biological nanomaterials through hydrogen bonds. For example, Lee et al. fabricated a ultrathin nanomesh membrane based on the self-assembly of M13 virus on graphene oxide (GO) nanosheets via both hydrogen bonds and electrostatic interactions [53]. In the self-assembly process of virus on the GO’s surface, the basic amino acids (histidine and lysine) of the virus enable strong electrostatic and hydrogen bond interactions with the carboxylate groups at the edges of GO nanosheets. In another case, Xue et al. investigated the binding of DNA with GO through the surface plasmon resonance (SPR) technique [54], and found that the hydrogen bond plays a key role in the interactions between single-stranded DNA (ssDNA) with GO, which enabled the fabrication of a novel biosensor for highly sensitive and selective determination of ssDNA targets.

#### 2.1.2. Electrostatic Interaction

Electrostatic interactions play significant roles in the self-assembly of peptides, proteins, enzymes, and others into higher hierarchical nanostructures and at the same time stabilize the formed nanostructures [55,56,57].

**Figure 2:**
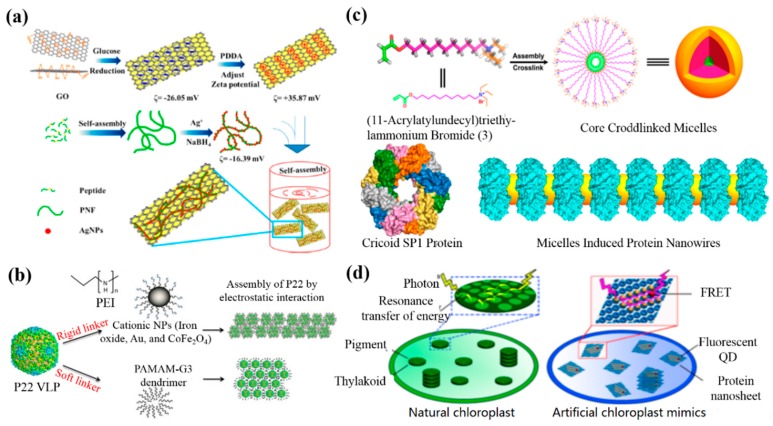
Electrostatic-interaction-mediated self-assembly of biomolecule-based nanomaterials: (**a**) The formation of peptide nanofiber (PNF)-bioinspired silver nanowires (AgNWs) on graphene nanosheets (GNs). Reprinted with permission from [58]. Copyright 2014 American Chemical Society. (**b**) The synthesis of self-assembled P22 virus-like particles (VLPs) via rigid (inorganic nanoparticles (NPs)) and soft (PAMAM) cationic linkers. Reprinted with permission from [59]. Copyright 2017 Materials Research Society. (**c**) The structure-based design of protein nanowires. Reprinted with permission from [60]. Copyright 2016 American Chemical Society. (**d**) Protein nanosheet–quantum dot (QD) nanohybrids. Reprinted with permission from [61]. Copyright 2017 American Chemical Society. PEI, polyethyleneimine; FRET, fluorescence resonance energy transfer.

Wang et al. investigated the self-assembly of a motif-designed peptide for the formation of peptide nanofibers (PNFs) and bioinspired PNF-based silver nanowires (AgNWs), and fabricated graphene nanosheet (GN)-PNF-AgNW nanocomposites through an electrostatic interaction between negatively charged PNF-AgNWs and a polymer-modified, positively charged GN [58]. Firstly, a positively charged GN was obtained with a zeta potential of +35.87 mV when a GN was functionalized with the positively charged polymer PDDA. Subsequently, the PNF-AgNW nanohybrids were created by controlling the self-assembly of a designed peptide molecule and subsequent bioinpired synthesis of AgNPs on PNFs, and the formed PNF-AgNW nanohybrids exhibited a zeta potential of −16.39 mV. Therefore, the PNF-AgNW nanohybrids were bound onto PDDA-GN by the electrostatic interaction easily, as indicated in Figure 2a.

Gupta and co-workers demonstrated the inorganic NP (CoFe_2_O_4_ and Au)-induced self-assembly of bacteriophage P22 via an electrostatic interaction [59]. In their work, negatively charged P22 virus-like particles (VLPs) were synthesized by the co-expression of the coat protein and scaffold protein in *Escherichia coli*. The positively charaged CoFe_2_O_4_ and Au NPs were prepared by coating the NPs with polyethyleneimine via a modified bilayer phase transfer method. Finally, the P22 VLPs were assembled through a controllable electrostatic interaction between the negatively charged VLP and the positively charged polymer-modified NPs, as shown in Figure 2b. In another study, Liu and co-workers fabricated micelle-induced protein nanowires via an electrostatic interaction when the electronegative cricoid stable protein one (SP1) assembled with positively charged core-crosslinked micelles (Figure 2c) [60]. Recently, they further used SP1 as a building block to assemble positively charaged semiconductor quantum dots (QDs) via an electrostatic interaction to obtain highly ordered protein nanowires with prominent optical properties (Figure 2d) [61].

#### 2.1.3. Hydrophobic Interaction

Many biomolecules, such as peptides and proteins, can form highly ordered self-assembled superstructures via a hydrophobic interaction due to their hydrophobic property [62]. It is well-known that amino acids can be divided into hydrophobic and hydrophilic ones due to their amino acid residues. To date, a lot of studies on the hydrophobic-interaction-induced self-assembly of proteins and peptides for functional bionanomaterials have been reported [63,64].

For instance, Liao et al. investigated the self-assembly mechanism of PNFs in solution and on a surface by using a small peptide amphiphile (PA) (NapFFKYp) as a model [65]. It was found that this PA first grows into nanofibers via a nucleation process, and then forms highly ordered nanofibers in solution by a hydrophobic interaction. However, the self-assembly of this PA could form mixed nanofiber and nanosheet structures on a substrate. Further molecular dynamics simulations (MDSs) suggested that both hydrophobic and ion–ion interactions are crucial during the self-assembly process of this PA. In another study, Yang and co-workers demonstrated how a model ionic-complementary peptide EAK16-II (AEAEAKAKAEAEAKAK) assembles on hydrophilic (mica) and hydrophobic (HOPG) substrates via electrostatic and hydrophobic interactions, respectively [66].

**Figure 3:**
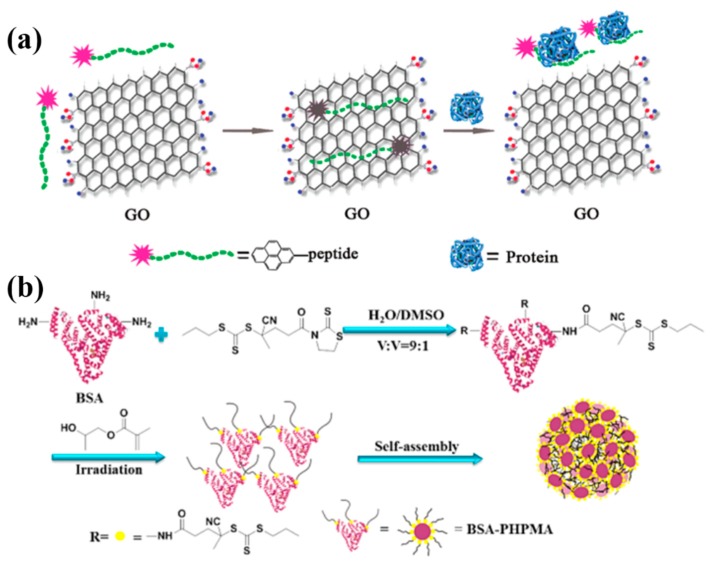
The hydrophobic interaction for biomolecular self-assembly: (**a**) A pyrene-labeled peptide for monitoring the protein–peptide interactions. Reprinted with permission from [71]. Copyright 2011 American Chemical Society. (**b**) Self-assembly of bovine serum albumin (BSA)-based nanoparticles to microspheres. Reprinted with permission from [72]. Copyright 2017 American Chemical Society. GO, graphene oxide.

Biomolecules can also be conjugated with other nanomaterials, such as graphene or NPs, to form functional nanomaterials via a hydrophobic interaction [67,68,69,70]. For example, Lu and co-workers developed a novel fluorescent approach to monitor peptide–protein interactions based on the assembly of a pyrene-labeled peptide on GO via both π–π and hydrophobic interactions [71]. To achieve the aim, the peptide was firstly modified with a pyrene group to form a π-rich framework with high fluorescence, and then the pyrene-labelled peptide was mixed with GO to obtain a GO-pyrene-peptide nanocomposite through both π–π and hydrophobic interactions, as shown in Figure 3a. Due to the peptide–protein interaction, the competitive binding of an antibody with GO for the pyrene-labelled peptide decreased the adsorption of the peptide on the GO and promoted the formation of a peptide–antibody complex in solution (Figure 3a).

In another study, Ma et al. demonstrated a facile strategy to prepare protein-based NPs [72], where bovine serum albumin (BSA) was modified with multi-photoinitiated reversible addition-fragmentation chain transfer (RAFT) polymerization to the BSA–PHPMA conjugates. The synthesized BSA–PHPMA conjugates were further aggregated into NPs through the hydrophobic interaction of PHPMA (Figure 3b). Zhang et al. reported the immobilization and self-assembly of horseradish peroxidase (HRP) and oxalate oxidase (OxOx) on chemically reduced graphene oxide (CRGO) [73]. Their results indicated that the enzymatic loading can be improved by increasing the reduction degree of GO, and the excellent properties of the CRGO–enzyme conjugates are attributed to hydrophobic interactions between enzymes and the CRGO’s surface. Studies on the self-assembly of proteins and enzymes on a material’s surface are helpful for designing and fabricating novel biosensors for the high-performance sensing of various analytes.

#### 2.1.4. π–π Interaction

Noncovalent π–π interactions are another potential driving force to promote the self-assembly of biomolecules [74,75]. Some biomolecules, including peptides, proteins, DNA, enzymes, and viruses, mostly contain aromatic motifs, making it possible to form highly ordered superstructures by π–π stacking or functional hybrids by biomolecule–material π–π interaction.

**Figure 4:**
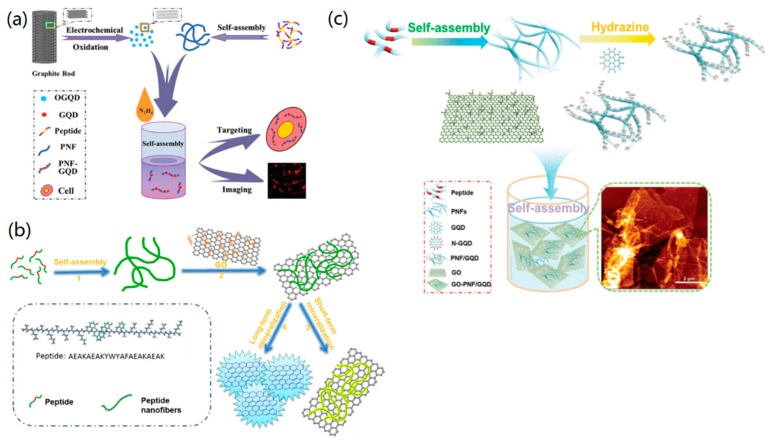
π–π-interaction-mediated self-assembly of nanomaterials: (**a**) The fabrication of PNF– graphene quantum dot (GQD) nanohybrids. Reprinted with permission from [76]. Copyright 2015 WILEY-VCH. (**b**) The synthesis of GO–PNF nanohybrids and GO-PNF-HA minerals. Reprinted with permission from [33]. Copyright 2015 Elsevier. (**c**) The synthesis of PNFs and binary GQD-PNF, and ternary GQD–PNF–GO nanohybrids. Reprinted with permission from [77]. Copyright 2017 WILEY-VCH.

For example, Su and co-workers investigated the self-assembly of a designed peptide (RGDAEAKAEAKYWYAFAEAKAEAKRGD) to PNFs and the π–π conjugation between PNFs and graphene quantum dots (GQDs) towards novel PNF–GQD nanohybrids for the simultaneous targeting and imaging of tumor cells [76]. The designed peptide has trifunctional motifs, in which RGD can recognize the integrin-rich tumor cells, the AEAKAEAK motif provides the capability of self-assembly and formation of PNFs, and the motif of YWYAF has the tendency to bind with GQDs via π–π interactions (Figure 4a). In another study, they synthesized GO–PNF nanohybrids via π–π interactions between designed PNFs and GO, and further utilized the formed GO–PNF nanohybrids as templates for the biomimetic mineralization of hydroxyapatite (HA) (Figure 4b) [33]. Recently, they also used the peptide (AEAKAEAKYWYAFAEAKAEAK) to synthesize GQD–PNF–GO nanohybrids via π–π interactions between the created PNFs with GQDs and GO [77], as shown in Figure 4c. To prove the unique interactions among the three components, they used the atomic force microscopy (AFM)-based force spectroscopy technique to measure the interactions (including π–π binding forces) between PNFs with GQDs and GO, and the obtained results showed that the rupture force between PNFs and GO was stronger than the force between GQDs and GO as well as between GQDs and PNFs.

Besides peptides, other biomolecules, such as proteins, DNA, enzymes, and viruses, have also been widely used to conjugate with graphene to form nanomaterials via π–π interactions for various applications [78,79]. For example, Wang and co-workers designed a uniform three-dimensional (3D) graphene-nanodots-encaged porous gold electrode for loading enzyme [80]. Pyrene-functionalized glucose oxidase (GOx) and catalase (CAT) were prepared and used as the immersed solution for the modification of the electrode. In the process of enzyme modification, Pyrene-GOx/CAT was loaded onto the graphene-nanodots-encaged porous gold electrode via π stacking between pyrene and graphene. The fabricated enzyme electrodes showed an excellent catalytic performance compared with native enzyme. Huang et al. designed a GQDs–ionic liquid–nafion (GQDs-IL-NF) composite film [81], which could interact with ssDNA through noncovalent π–π interactions to fabricate a novel biosensor platform for detecting a carcinoembryonic antigen with high sensitivity.

By using the π–π interaction between ssDNA and RGO, Li et al. created ssDNA–RGO composites for the further bioinspired synthesis of cotton-flower-like platinum nanoparticles (PtNPs) [82]. The ssDNA molecules were conjugated with RGO via the π–π interaction and PtNPs were formed on the surface of ssDNA–RGO by the bioinspired synthesis. The created ssDNA-RGO-PtNPs material exhibited high catalytic activity for methanol oxidation and CO tolerance.

### 2.2. Biomolecular-Specific Interactions

#### 2.2.1. DNA/RNA Base Pairing

The focus of DNA/RNA nanotechnology is to direct ssDNAs/ssRNAs to self-assemble into desired 1D, two-dimensional (2D), and even 3D nanomaterials via DNA/RNA base pairing.

**Figure 5:**
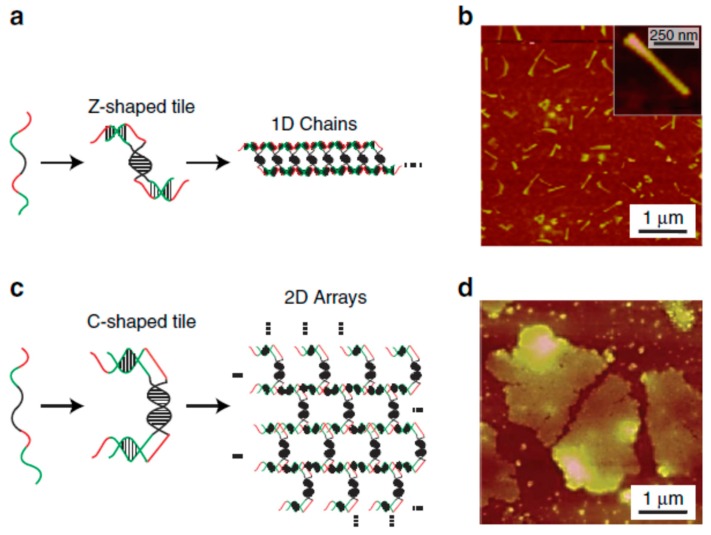
The in vitro genetic-encoding-mediated self-assembly of DNA to different nanostructures: (**a**,**b**) nanowires and (**c**,**d**) two-dimensional (2D) nanosheets. Reprinted with permission from [85]. Copyright 2016 Macmillan Publishers Limited. 1D, one dimensional.

Previously, it has been reported that DNA nanostructures could be created by controlling the DNA base pairing through several methods, including the clamped hybridization chain reaction [24], paranemic crossover DNA motif assembly [83], DNAzyme-based logic gate [84], and genetic encoding [85]. The basic principles for creating functional DNA nanomaterials are based on the design of DNA motifs/tiles and the subsequent controllable DNA base pairing. In a typical study, Elbaz and co-workers investigated the fabrication of DNA nanostructures by guiding the self-assembly of gene-encoded DNA in living bacteria [85]. To achieve this aim, a short ssDNA was first encoded as a gene and then enzymatically converted to a new ssDNA. This in-vivo-produced ssDNA could be utilized for in-vitro fabrication of 1D nanowires (Figure 5a,b) and 2D nanosheets (Figure 5c and d). To form a Z- and C-shaped tile, two red pairs of a symmetric motif hybridized with two other red pairs of another symmetric motif. The motif with a short black domain mediated the formation of the Z-shaped (Figure 5a) tile and the motif with a long black domain promoted the formation of the C-shaped tile (Figure 5c). This work provided a novel strategy to prepare functional DNA nanostructures for in-vivo applications.

Besides the simple DNA nanostructures, DNA nanotechnology provides the possibility for the programmable design and synthesis of complex nanostructures, such as DNA origami [86,87] and nanoswitches [88]. For instance, in the DNA origami technique, long ssDNA is folded into target shapes by using short DNA staples, which are designed to be complementary to particular regions of the long DNA [86]. Therefore, a lot of 2D and 3D DNA nanostructures can be created by using the further self-assembly of DNA origami and nanoswitches [86,87], which can serve as nanoscale templates to form biomolecule and NP-based nanomaterials [89,90].

Similar to DNA base pairing, RNA can also be assembled into various nanostructures (such as tetrahedrons, nanotriangles, lattices, and tubes) by bottom-up self-assembly based on intra- and inter RNA interactions [91,92,93]. These self-assembled RNA nanostructures have shown wide applications for drug or NPs delivery and cancer diagnostics [91,93].

Previously, Gazit and co-workers have prepared self-assembled peptide nucleic acid (PNA) fibers with a unique light-emitting property [94], in which the self-assembly of PNA is related to both π–π stacking interactions and Watson–Crick base pairing.

#### 2.2.2. Ligand–Receptor Binding

It is possible to utilize specific molecule–molecule recognitions, such as the ligand–receptor [95,96,97] and antigen–antibody [98,99] bindings, to guide the self-assembly of biomolecules to form ordered nanostructures and nanomaterials.

**Figure 6:**
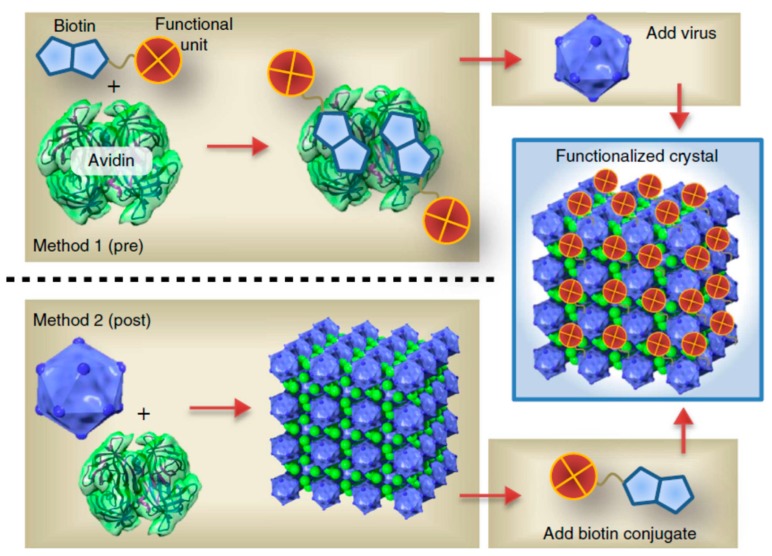
The avidin–biotin-binding-mediated self-assembly of a protein cage to three-dimensional (3D) functional crystals. Reprinted with permission from [95]. Copyright 2014 Macmillan Publishers Limited.

Liljeström et al. investigated the self-assembly and modular functionalization of 3D cowpea chlorotic mottle virus (CCMV) crystals by using the avidin–biotin recognition [95]. The functionalization and self-assembly of 3D CCMV crystals were achieved in two ways, as shown in Figure 6. In the first way (Method 1), the functionalization was achieved by first modifying avidin with biotin-linked functional units (such as dyes, enzymes, or NPs), and then adding CCMV particles to form 3D crystals through self-assembly. In another method (Method 2), the mixing of CCMV and avidin together caused the formation of 3D crystals via self-assembly, which could be further functionalized with biotin-linked functional units to form functional 3D nanomaterials. The use of avidin–biotin binding in this study allowed for the highly selective functionalization of protein crystals, which exhibited great potential for biomedical applications. In another study, Xu and co-workers demonstrated that a ligand–receptor interaction could modulate the energy landscape of peptide self-assembly and affect the formation of various nanostructures [96]. Their study proved that it is possible to use a ligand–receptor interaction to modulate the kinetics of enzyme-mediated peptide self-assembly.

The formation of antigen–antibody immunocomplexes is helpful for the molecular self-assembly and the formation of functional bionanomaterials. For instance, Kominami and co-workers investigated the self-assembly of immunoglobulin G (IgG) on a mica surface, and found the formation of 2D hexameric IgG crystal [99]. Therefore, the antigen (anti-human serum albumin) could be bound onto the IgG hexamer via the antigen–antibody binding.

#### 2.2.3. Biomolecule–Polymer Conjugates for Self-Assembly

The conjugation between biomolecules (such as a lipid and cholesterol) with polymers can also mediate the self-assembly of the formed conjugates to various nanostructures [100,101]. For instance, the mixing of lipid particles with amphiphilic, hydrophobic, and hydrophilic drugs for the formation of hybrid cubosomes and hexosomes has exhibited great potential for advanced drug delivery systems [100,102,103].

Cholesterol, one of the important biopolymers, has been utilized as a versatile building block to conjugate with various polymers for the fabrication of self-assembled functional nanomaterials [104,105,106]. For example, Yang and co-workers demonstrated the preparation of polyoxometalate–cholesterol conjugates, which could self-assemble into microrods and nanoribbons by controlling the reaction temperature [104]. Engberg et al. found that cholesterol could be tethered into poly(ethylene glycol) (PEG) networks, via polymerization in an organic solvent, that were capable of forming weakly ordered aggregates via self-assembly [105].

## 3. External Stimulations towards Biomolecular Self-Assembly

The self-assembly of biomolecules is highly sensitive to, among other things, the molecular structure, the solution environment, including the pH, temperature, and ionic strength, organic solvents, and enzymes [107,108]. In this part, we will introduce and discuss the potential strategies for promoting the self-assembly of various biomolecules.

### 3.1. pH Effect

**Figure 7:**
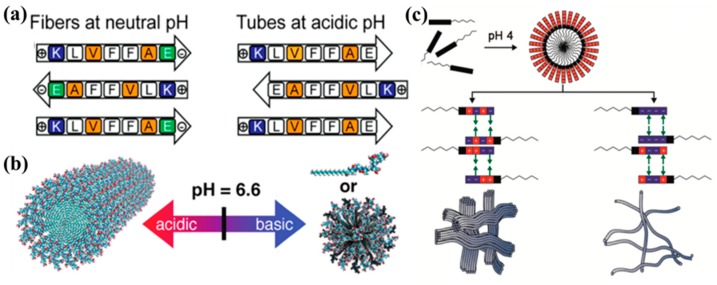
The pH effect on the self-assembly of biomolecules: (**a**) Self-assembled fibers and tubes under different pH conditions. Reprinted with permission from [112]. Copyright 2017 American Chemical Society. (**b**) The pH-triggered morphological transition of self-assembling PA. Reprinted with permission from [113]. Copyright 2012 American Chemical Society. (**c**) Self-assembled nanofibers by pH-mediated lateral assembly. Reprinted with permission from [114]. Copyright 2015 American Chemical Society.

The growth of biological nanostructures is also influenced by the solution conditions, such as pH, temperature, and ionic strength [109,110,111]. For instance, the peptide sequence KLVFFAE from the Aβ (16–22) peptide of Alzheimer’s (AD) is very sensitive to the environmental pH. Hsieh and co-workers demonstrated that the Aβ (16–22) peptide (KLVFFAE) could self-assemble in neutral and acidic conditions to different nanostructures [112]. Under a neutral condition, the peptide assembled into nanofibers due to the cross-strand pairing between the positively charged K_16_ and the deprotonated C-terminal E_22_ side chain. However, under an acidic pH condition, the peptide formed nanotubes because the protonated E_22_ side chain weakened the K_16_–E_22_ salt bridge, and the strands shifted out of register and grew into nanotubes (Figure 7a). In another example, Ghosh et al. developed a strategy for precisely tuning the self-assembly behavior of PA by adjusting the solution pH [113]. They found that PA could self-assemble into nanofibers under pH 4 and spherical nano-micelles at pH 10 (Figure 7b). Furthermore, Chen et al. designed a pH-controlled system that could control the PA self-assembly into micelles, nanofibers, and nanofiber bundles due to the clever designs of complementary electrostatic attraction using oppositely charged amino acid pairs, such as arginine and aspartic acid, when the pH was changed (Figure 7c) [114].

The self-assembly of proteins, enzymes, and viruses is also affected by the solution property [115,116]. For instance, Brodin et al. utilized a Rosetta-interface-designed cytochrome 3 (RIDC3) to self-assemble 1D protein nanotubes and 2D protein nanosheets through Zn^2+^-coordination [117,118]. In their studies, the morphology of the self-assembled RIDC3 was dependent on the concentration of Zn^2+^, the RIDC3 concentration, and the pH of the solution. The Zn^2+^-coordination was reduced when the pH and ([Zn]:[RIDC3]) ratios were decreased to lower conditions; thus, the formed 1D microtubes were transferred into 2D nanosheets due to the reduced nucleation efficiency of Zn-mediated RIDC3 [117]. In addition, 2D Zn^2+^-RIDC3 arrays were formed under the condition of a lower concentration of Zn^2+^ and a lower pH [118].

The self-assembly of DNA molecules to ordered nanostructures can also be mediated by the pH-responsive formation of a triplex/tetraplex [119,120,121]. For instance, Wu and Willner recently reported the pH-stimulated reconfiguration and structural isomerization of a DNA origami dimer and trimer by designing pH-sensitive origami dimers and trimers [121]. It was known that triplex DNA nanostructures containing Hoogsteen-type C-G·C^+^ bridges can be stabilized under acidic conditions and then separated under neutral systems, while the triplex strands, including T-A·T bridges, can be stabilized at neutral pH and then separated under basic systems. On this basis, they performed the pH-stimulated cyclic assembly and separation of the oligomeric origami, and proved the programmed site-specific cleavage of trimer–origami and the reassembly of the separated units. In addition, the pH-mediated isomerization of a linear three-frame origami into a bent configuration has also been proved.

### 3.2. Temperature Effect

It is well-known that temperature is another important factor that affects the conformation and the intermolecular interactions of biomolecules in solution [122,123].

**Figure 8:**
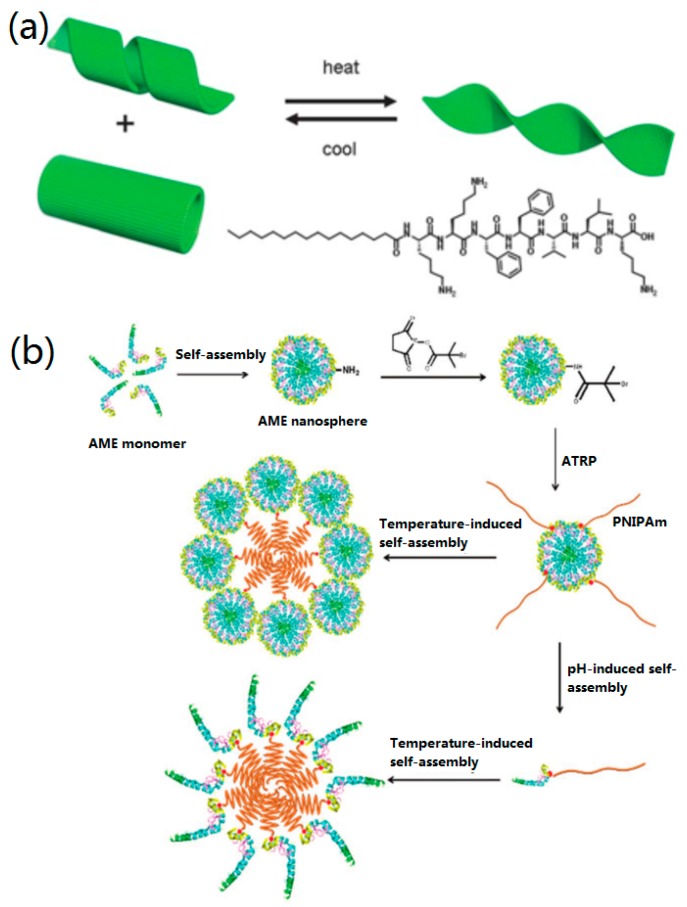
The temperature effect on biomolecular self-assembly: (**a**) The thermo-reversible transition and (bottom right) structure of PA. Reprinted with permission from [125]. Copyright 2013 The Royal Society of Chemistry. (**b**) The synthesis and proposed model of self-assembly and disassembly of pH- and temperature-responsive Amelogenin (AME)–PNIPAm bioconjugates. Reprinted with permission from [126]. Copyright 2018 WILEY-VCH.

Previously, Hamley’s group found that the PA palmitoyl-KTTKS showed a thermal transition from nanotapes to micelles when the temperature was changed from 20 to 30 °C [124]. In a further study, they studied the effect of temperature on the self-assembly mechanism of PA (C16-KKFFVLK) [125]. They used cryogenic transmission electron microscopy (cryo-TEM), small-angle X-ray and scattering, and circular dichroism (CD) spectra to observe the reversible thermal transition and self-assembly of PA. It was found that PA self-assembled into nanotubes and helical ribbons at room temperature. Interestingly, PA self-assembled into twisted tapes under heating, but the nanotubes and ribbons were reformed under cooling, as shown in Figure 8a.

The self-assembly of polymers and proteins by controlling the temperature has also been studied [101,102,103,104]. For instance, a protein–polymer biohybrid was designed by controlling both temperature and pH [126]. Firstly, a hydrophilic initiator (2-bromoisobuta-noic acid N-hydroxysuccinimide (NHS-BiB)) was immobilized onto the surface of an Amelogenin (AME) nanosphere to form a macroinitiator (AME initiator), and then poly(N-isopropylacrylamide) (PNIPAm) chains were grafted to form AME–PNIPAm bioconjugates by temperature-induced self-assembly (Figure 8b). When the temperature was increased from 20 to 40 °C, the hydrodynamic particle size was increased to 218.7 nm with a very narrow size distribution. Huang et al. designed a “rod-coil” graft copolymer containing a polyphenylene backbone linked with poly(ethylene oxide) (PEO) side chains [127], which could form nanoribbons and multilayer sheets at different temperatures.

In another example, the effect of incubation temperature on the self-assembly of regenerated silk fibroin (RSF) was investigated by Zhong and co-workers [128]. They found that the effect of temperature on the self-assembly of RSF was dependent on the concentration of RSF. For a relatively low concentration of RSF, the increase in incubation temperature promoted the formation of anti-parallel β-sheet protofibrils and inhibited the growth of random coil protofilaments/globule-like molecules. However, under a higher concentration of RSF, the increase in incubation temperature changed the morphologies of RSF from protofilaments to protofibrils and beads, and then to longer nanofibers and globules. This work makes it clear that the conformation and morphology of biomolecules can be tuned by controlling the incubation temperature, which will be helpful for us to understand the formation mechanism of various RSF-based biomaterials and extend their biomedical applications.

### 3.3. Ionic Effect

Along with the effects of pH and temperature, the effect of ions/ionic strength on the self-assembly of proteins [129,130], peptides [131], DNA [132], and RNA [133] molecules have been reported.

Semerdzhiev et al. investigated the self-assembly of α-synuclein into protein fibrils and suprafibrillar by adjusting some external stimulations, including pH, temperature, ionic strength, protein concentration, and seeding [130]. Their results indicated that the formation of suprafibrillar protein assemblies requires a high salt concentration (>10^4^ µM K^+^/Na^+^). However, at a low ionic strength (about 10^2^ µM K^+^/Na^+^), the creation of individual protein fibrils was dominant in the solution due to the strong interfibril repulsion. With the increasing of salt concentration, the electrostatic effects between protein fibrils were screened, promoting the interactions between the formed fibrils and the formation of a sheet-like structure. Further increasing the ionic strength even caused the formation of cylindrical protein aggregates.

Dai and co-workers reported the tunable assembly of an amyloid-forming peptide towards nanosheet structures [131]. They found that the size and yield of the self-assembled amyloid peptide (KLVFFAK) nanosheets could be fine-tuned by adjusting the ionic strength in aqueous solution. With the increasing of NaCl concentration from 0.1 to 1.0 M, the width and the yield of self-assembled peptide nanosheets increased accordingly. They suggested that salt could improve the aggregation ability of peptide molecules by screening out the repulsive interactions between the positively charged Lys-Lys contacts.

**Figure 9:**
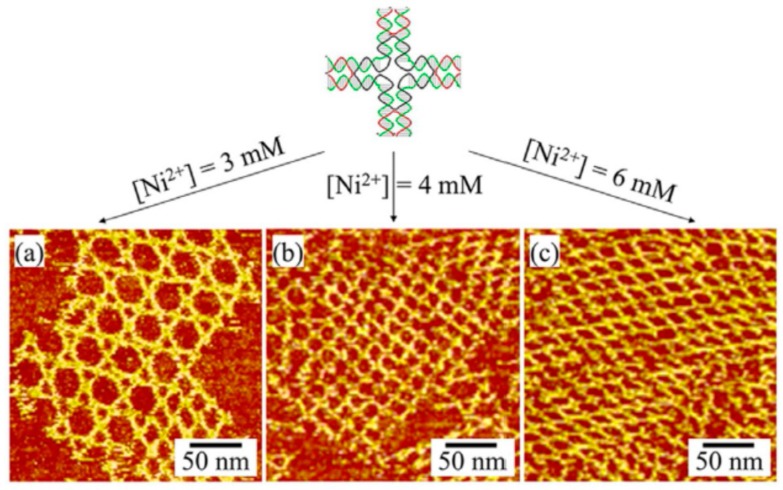
Self-assembled DNA nanostructures on a mica surface by adjusting the Ni^2+^ ion concentration: (**a**) 3, (**b**) 4, and (**c**) 6 mM. Reprinted with permission from [132]. Copyright 2017 Wiley-VCH.

The self-assembly of DNA and RNA is also affected by the ions and ionic strength. For instance, Liu and co-workers demonstrated the self-assembly of DNA on a mica surface [132], which can be regulated by changing the concentration of Ni^2+^ to form a salt bridge between DNA and the mica surface. They found that a suitable Ni^2+^ concentration was crucial for the formation of 2D DNA arrays on the mica surface. A low Ni^2+^ concentration did not provide enough attractive force to bind DNA to the mica surface, while an Ni^2+^ concentration that was too high caused a strong DNA–surface attraction and hindered the DNA mobility and self-assembly. AFM experiments indicated that 2D DNA trihexagonal (Figure 9a), square (Figure 9b), and rhombic (Figure 9c) arrays were formed by controlling the self-assembly of the four-pointed-star DNA motif via adding 3, 4, and 6 mM Ni^2+^, respectively. This study proved that the weak DNA–DNA interactions could be stabilized by using a suitable ionic concentration to regulate the DNA–surface interactions, promoting the formation of larger DNA nanostructures. Recently, Yang and co-workers demonstrated a novel K^+^ ion-stimulated self-assembly of DNA origami nanostructures by using G-quadruplexes as stimuli-responsive bridges [134]. It was found that, with the stimulation of monovalent cations, the conformation transitions between the G-quadruplex and its sing-strand state promoted the reversible assembly process of DNA origami. Their study provides a potential strategy for designing pH-responsive DNA nanomaterials for biomedical and nanotechnological applications. In another study, Garmann et al. studied the assembly pathway of an icosahedral ssRNA virus and found that the in-vitro assembly of ssRNA virus was affected by the pH and ionic strength [133].

### 3.4. Organic Stimulators

It is known that the structural formation of self-assembled biological nanomaterials is related to the intermolecular noncovalent interactions, including hydrogen bonds, electrostatic interactions, hydrophobic interactions, and π–π interactions. However, the conformation transition and self-assembly of biomolecules are affected by some organic solvents due to the synergistic effects with these interactions.

**Figure 10:**
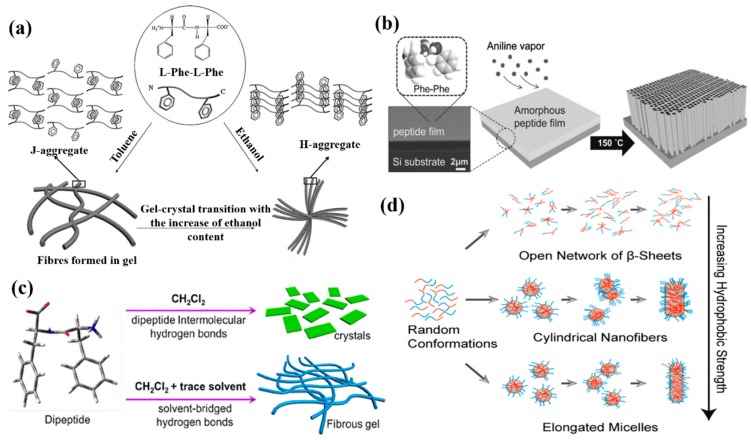
Effects of organic solvents on biomolecular self-assembly: (**a**) The structural transition of FF nanofibers in mixed organic solvents. Reprinted with permission from [136]. Copyright 2010 WILEY-VCH. (**b**) Vertically well-aligned peptide nanowires prepared by high-temperature aniline vapor aging. Reprinted with permission from [140]. Copyright 2008 WILEY-VCH. (**c**) A phase transition induced by trace amounts of organic solvent. Reprinted with permission from [142]. Copyright 2016 American Chemical Society. (**d**) Kinetic mechanisms of peptide self-assembly studied by molecular dynamics simulation (MDS). Reprinted with permission from [143]. Copyright 2015 American Chemical Society.

The self-assembly of peptides and proteins into various morphologies in different organic solvents have been investigated widely [135,136,137,138,139,140,141]. For instance, Yan et al. investigated the self-assembly of a small FF peptide in chloroform and toluene, and found that the peptide can self-assemble into long nanofibrils and then entangle to form organogels [135]. It was found that the created FF-based organogels were thermo-responsive and the sol-gel process was thermo-reversible. In a further study, they investigated the effects of organic co-solvents (ethanol and toluene) on the stabilization of the created organogels [136]. Flower-like microcrystals were prepared by a further self-assembly process of gels (Figure 10a). The solvent (ethanol) has a higher polarity than toluene, and, therefore, it caused the formation of hydrogen bonds during biomolecular self-assembly and promoted the subsequent transition of organgels to microcrystals.

Ryu et al. investigated the high-temperature-induced self-assembly of an FF peptide into vertically aligned nanowires in the environment of aniline vapor [140]. In their work, an FF peptide solution dissolved in 1,1,1,3,3,3-Hexafluoro-2-propanol (HFIP) was first dropped onto a silicon wafer or quartz plate substrate. Subsequently, the peptide solution was dried in a vacuum desiccator to form a patterned FF film. Finally, the FF film was aged under an aniline vapor condition at 150 °C to obtain vertical peptide nanowire arrays on a silicon substrate, as shown in Figure 10b.

Recently, Wang et al. found that a trace amount of solvent can be a predominant factor to control the self-assembly of an FF peptide in dichloromethane, ethanol, *N,N*-dimethylformamide (DMF), and acetone [142]. They demonstrated that hydrogen bonding plays more of a role in the process of nanofiber formation than other noncovalent interactions (Figure 10c), and that the bonding of C=O and N–H in FF molecules was affected by the used organic solvents.

To further understand the effects of organic solvents on the self-assembly of peptides, Fu et al. used molecular dynamics simulations (MDSs) to study the solvent effects on the self-assembly of PAs [143]. When the hydrophobic interaction was weak, biomolecular aggregates were formed due to the hydrophobic interaction and hydrogen bonding. In addition, the aggregates could grow with different directions, resulting in the formation of an open network structure. However, the structure was changed from open one to a closed one when the hydrophobic interaction was increased. When the hydrophobic interaction was further increased, the hydrogen bonds were reduced and all of the peptides appeared in a random-coil conformation and formed an elongated micelle structure, as indicated in Figure 10d.

The above examples and discussions provide experimental and theoretical evidence that organic solvents exert significant effects on the conformation transition and self-assembly pathways of biomolecules.

### 3.5. Enzymatic Stimulators

Enzymes can also influence the self-assembly of biomolecules significantly as they may catalyze the formation of biological materials. Based on the functions and types, enzymes can promote or inhibit the aggregation and self-assembly of biomolecules [144]. In recent decades, many studies on enzymatic driving for the formation of NPs, nanofibrils, crosslinked hydrogels, and other superstructures have been reported [145,146].

**Figure 11:**
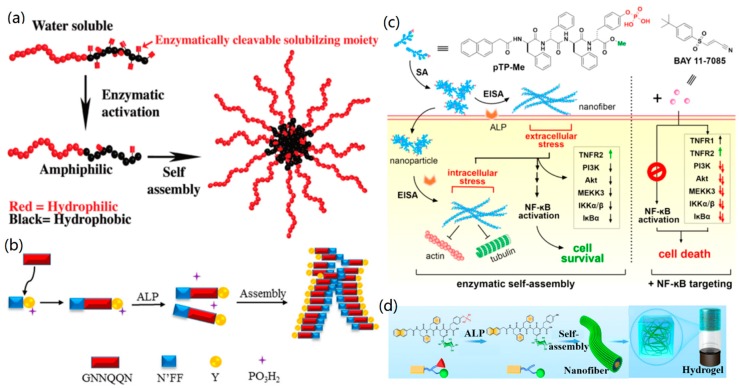
The enzyme-mediated self-assembly of biomolecules: (**a**) The enzyme-active self-assembly of water-soluble diblock copolymers to colloidal nanostructures. Reprinted with permission from [147]. Copyright 2009 American Chemical Society. (**b**) Alkaline phosphatase (ALP)-mediated formation of a peptide hydrogel. Reprinted with permission from [152]. Copyright 2016 The Royal Society of Chemistry. (**c**) The enzyme-induced self-assembly of pTP-Me into PNFs. Reprinted with permission from [155]. Copyright 2018 American Chemical Society. (**d**) The self-assembly of a glycopeptide to a supramolecular hydrogel. Reprinted with permission from [156]. Copyright 2018 American Chemical Society.

For example, Amir et al. introduced a novel enzyme-triggered strategy to mediate the self-assembly of a block coplymer into NPs under a physiological condition [147]. In their work, they designed a water-soluble diblock copolymer containing a hydrophilic block copolymer and a block of phosphorylated 4-hydroxystyrene, and then the phosphate groups of the copolymer were removed by phosphatase to form the amphiphilic diblock copolymers. Subsequently, the amphiphilic copolymers were self-assembled into colloidal NPs via an in-situ process (Figure 11a). This approach constitutes a new way to form polymeric materials by using various polymeric backbones and enzymatic triggers.

Besides biopolymers, the self-assembly of other biomolecules, such as proteins, peptides, and DNA, could be driven by enzymes [148,149,150,151]. Previously, Xu’s group used the enzyme-instructed self-assembly (EISA) approach to prepared a few supramolecular nanostructures, such as nanofibers and hydrogels [13,152,153,154]. For example, they designed a series of structural precursors based on the peptide GNNQQNY sequence of the yeast prion Sup35, which can self-assemble to form supramolecular hydrogels induced by alkaline phosphatase in water (Figure 11b) [152]. In another study [155], they investigated the enzyme-induced in-situ self-assembly of C-terminal methylated phosphotetrapeptide (pTP-Me) into PNFs. It was found that the obtained PNFs exhibited strong synergism with NF-κB targeting for the selective necroptosis of cancer cells (Figure 11c).

Qi and co-workers developed a novel hydrogel from the enzyme-induced supramolecular self-assembly of a synthetic glycopeptide to mimic the glycosylated microenvironment of the extracellular matrix [156]. In their work, a gelator precursor 1 glycopeptide, containing a naphthyl group, a tetrapeptide motif (Phe-Phe-Asp-Tyr(H_2_PO_3_)), and a sugar moiety (D-glucosamine), was first prepared via a solid-phase synthesis method. Then, the glycopeptide was dissolved in water with a pH value of 7.4 and transferred into gelator 2 by adding alkaline phosphatase (owing to the enzymatic dephosphorylation towards gelator 1). After that, the gelator self-assembled into nanofibers and then into a hydrogel at room temperature via aromatic–aromatic and hydrogen bonding interactions, as shown in Figure 11d. Furthermore, the fabricated hydrogel could serve as biomimetic scaffold to promote the generation of new blood capillaries in vitro and vivo.

### 3.6. Photo-Stimulation

Various biological nanostructures can also be obtained by the photo-induced self-assembly of biomolecules, such as peptides [157,158] and DNA [159,160,161]. The photo-triggered assembly of biomolecules exhibits a few advantages, such as reversibility, rapidity, remoteness, and cleanliness. In the photo-triggered assembly process, the photo-responsive groups acted as photoswitching units to mediate the structure and functions of the formed nanostructures.

Previously, Muraoka et al. synthesized photo-responsive PAs with a palmitoyl tail, the 2-nitrobenzyl group, and an oligopeptide motif (GV_3_A_3_E_3_), which were capable of self-assembling into supramolecular quadruple nanofibers [157]. Under the irradiation of light at 350 nm, the 2-nitrobenzyl group was cleaved, which dissociates the quadruple helical fibers to single non-helical fibrils. Ma and co-workers designed a photoswitchable molecule that can co-assemble with a cationic FF peptide to form elongated nanoplates and helical nanobelts under visible light [158]. After UV irradiation, the photo-isomerization of the photoswitchable molecule caused the disassembly of peptides to vesicle-like structures.

Similar to peptides, DNA molecules can also be induced by light stimulation to form self-assembled nanostructures. For instance, Tanaka and co-workers demonstrated the robust and photo-controllable synthesis of DNA structures (three-point-star motifs and capsules) by UV irradiation to the azobenzenes that were inserted into the sticky ends of DNA motifs [159]. Without the UV irradiation, the three-point-star motifs with azobenzenes self-assembled to sphere-shaped capsules, which were broken down into three-point-star motifs after UV irradiation for 50 s. Their study enhanced the potential of self-assembled DNA nanomaterials for controllable biomedical applications, such as precise drug delivery. Sugiyama and co-workers presented the fabrication of predesigned multiorientational patterns by photo-induced self-assembly of DNA origami nanostructures [160]. Firstly, they designed a series of 50-nm-sized hexagonal DNA origamis, which were then functionalized with photo-responsive oligonucleotides. Under visible light irradiation, the DNA origami self-assembled into predesigned oligomeric nanostructures, which could then disassemble into DNA origami structures under optimal UV irradiation at 40 °C. In a further study, they investigated the in-situ dynamic assembly/disassembly processes of photo-responsive DNA origami nanostructures, which can be placed on a lipid membrane surface [161]. It was found that the bilayer-placed DNA hexagonal structure was disassembled into monomers under UV irradiation, and reassembled into a larger DNA dimer after visible light irradiation.

All of the above cases prove the feasibility of photo-stimulation in the control of biomolecular self-assembly and the formation of various nanostructures.

### 3.7. Tailoring Molecular Structure

Molecular structure is crucial for guiding the self-assembly of biomolecules (especially for peptide and DNA molecules) into well-ordered superstructures [162,163]. For instance, by designing peptide sequences with multiple functions, such as recognition, binding, signal acceptor, and self-assembly motifs, it is easy to create 1D, 2D, and 3D peptide superstructures with tailored functions [8]. Meanwhile, it is possible to synthesize DNA superstructures by designing DNA sequences and other complex DNA building blocks [163].

Dai et al. used an amyloid peptide to self-assemble 2D peptide nanosheets (PNSs) by adjusting the molecular structure [131]. They changed the peptide sequence KLVFFAK into KLVFGAK and VQIVAK to provide the possibility of a β-sheet along the zippering axis face-to-face with the back pattern, and the three peptide sequences (KLVFFAK, KLVFGAK, and VQIVAK) could self-assemble into 2D nanosheets and nanofibers, respectively. Hence, the molecular structure of the peptide is very important for self-assembly into a well-ordered structure. In another case, Sun et al. discussed the self-assembly behaviors of three designed RADA16-1 peptides by studying the effects of motifs, pH, and assembly time [162]. Three functional peptide motifs, IKVAV, RGD, and YIGSR, were utilized to modify the RADA 16-1 peptide to provide different net charges and amphiphilic properties of the designed peptides at neutral pH. The obtained results indicated that both the electrostatic and hydrophilic/hydrophobic interactions of the motifs affected the self-assembly of the peptide and the morphologies of the formed PNFs.

**Figure 12:**
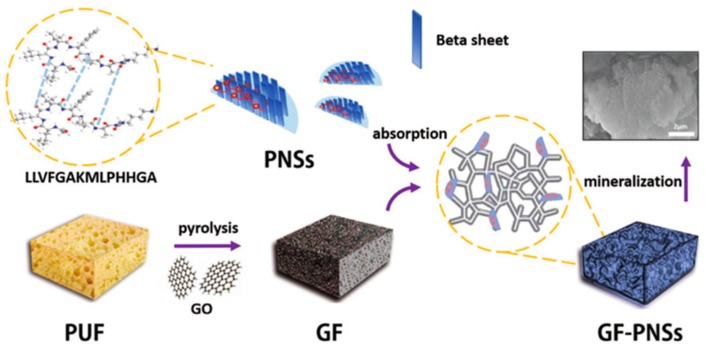
Two-dimensional peptide nanosheets (PNSs) by molecular tailoring: a schematic presentation of 2D peptide self-assembly and the biomimetic fabrication of 3D graphene foam (GF)-PNS-HA minerals. Reprinted with permission from [164]. Copyright 2018 WILEY-VCH.

Wei et al. designed several peptides with various functional motifs for the creation of functional 1D PNFs towards biomineralization, sensors, and cell targeting [33,76,77]. Very recently, they designed a novel peptide sequence (LLVFGAKMLPHHGA) to create 2D functional PNSs, as shown in Figure 12 [164]. The results indicated that the motif of LLVFGAK was responsible for the self-assembly and formation of PNSs, and KMLPHHGA provided an additional function for the biomineralization of HA. Therefore, the designed bifunctional PNSs exhibited unique properties for binding with 3D graphene foam (GF) to fabricate 3D biominerals.

Here, it is highly recommended for the authors to read two recent review papers on the design of small bioactive [165] and protein-mimic peptides [9] for biomaterials design and biomedical applications.

## 4. Various Self-Assembled Biological Nanostructures/Materials

Based on the above discussion, it can be concluded that hydrogen bonds, electrostatic interactions, hydrophobic interactions, and π–π interactions play important roles in mediating the self-assembly of biomolecules and promoting the formation of biological nanostructures. Other factors, such as molecular structure, pH effect, temperature, organic stimulators, and enzymatic stimulators are crucial for the self-assembly of biomolecules. To make it more clear, we summarize the types, nanostructures, interactions, and effect factors of the self-assembly of various biomolecules in Table 1.

## 5. Conclusions and Outlooks

The self-assembly of biomolecules provides a direct and effective way to create functional nanostructures and nanomaterials. Our deep understanding of the self-assembly mechanisms of biomolecules makes it possible to design and synthesize many novel biological nanomaterials with specific functions. In this review, we demonstrated the self-assembly of biomolecules into pure and hybrid biological nanomaterials from two perspectives (internal interactions and external stimulations) by introducing and discussing relevant cases. This work will be helpful for readers to understand basic methods to promote the self-assembly of biomolecules, develop novel biological nanomaterials, and explore the potential applications of self-assembled biological nanomaterials in materials science, biomedical engineering, tissue engineering, analytical science, and the energy and environmental sciences.

Biomolecular self-assembly towards functional nanomaterials has been one of the most focused-on fields in the last few years. In our opinion, the further development in this research field may include the following. First, the design of functional motifs for creating functional nanomaterials via self-assembly could be further studied. For example, the design of peptide molecules by combining a few functional motifs will form 1D to 3D nanostructures with multiple functions via peptide self-assembly. The design of DNA motifs can create uniform DNA nanostructures from nanowires to nanosheets and microcrystals through DNA hybridization. Second, it is important to develop bioinspired synthesis strategies by using self-assembled biological nanostructures to fabricate functional hybrid nanomaterials [166]. For instance, the conjugation between biological nanostructures and bioinspired NPs, QDs, and biominerals (such as HA and CaCO_3_) could introduce new properties and functions to the designed hybrid nanomaterials. Third, extensions to the applications of the self-assembled and bioinspired nanomaterials should be explored. More attention should be paid to the fabrication of energy storage materials and environment-related materials or techniques (such as filters, membranes, and sensing techniques).

## Figures and Tables

**Table 1:** A summary of the formed nanostructures via biomolecular self-assembly, and the internal interactions as well as external stimulations.

Biomolecules	Nanostructures	Interactions	Stimulations	Ref.
**Proteins**				
SP1	Nanowires	Electrostatic	Micelles	[60]
SP1	Nanowire-QDs	Electrostatic	Enzyme	[61]
BSA	NPs	Hydrophobic	Organic	[72]
IgG	2D crystals	Ligand–receptor	-	[99]
RIDC3	Nanotubes/2D Crystals	Zn^2+^-coordination	pH	[117,118]
Amelogenin	Nanospheres	-	pH and temperature	[126]
Silk fibroin	Protofibrils/Fibers	-	temperature	[128]
A-synuclein	Fibrils	Electrostatic	Ions	[130]
**Peptides**				
FF	Fibers/Tubes/Rods	Hydrogen bonds	Organic	[21]
FF	PNWs-G	Hydrogen bonds and π−π interaction	Organic	[52]
VIAGASLWWSEKLVIA	GN-PNF-AgNW	Electrostatic	Ethanol	[58]
NapFFKYp	Nanofibers	Hydrophobic	Organic	[65]
EAK 16-II	Nanofibers	Electrostatic/hydrophobic	Molecular structure	[66]
RGDAEAKAEAKYWYAFAEAKAEAKRGD	PNF-GQDs	π–π/Electrostatic	ethanol	[76]
AEAKAEAKYWYAFAEAKAEAK	GO-PNF	π–π/Electrostatic	Ethanol	[33]
AEAKAEAKYWYAFAEAKAEAK	GQD-PNF-GO	π–π/Electrostatic	Ethanol	[77]
Peptide	Fibers/Aggregates	Ligand–receptor	Enzyme	[96]
KLVFFAE	Nanofibers/Tubes	Electrostatic	pH	[112]
PA	Micelles/Nanofibers	Electrostatic	pH	[114]
C_16_-KKFFVLK	Nanotubes/Helical ribbons	Hydrogen bonds	Temperature	[125]
KLVFFAK	Nanosheets	Electrostatic	Ionic strength	[131]
GNNQQNY	Hydrogels	Hydrogen bonds	Enzyme	[152]
FFDY(H_2_PO_3_)	Fibers/Hydrogels	π–π/Hydrogen bonds	Enzyme	[156]
GV3A3E3	Fibers	Hydrogen bonds/hydrophobic	Light	[157]
FF	Nanoplates/belts	Hydrogen bonds/π–π	Light	[158]
**DNA/RNA**				
DNA	GQDs-ionic liquid (IL)-NF-DNA	π–π interactions	Enzyme	[81]
DNA	GO-DNA	π–π interactions	Temperature	[82]
DNA	Hydrogels	Clamped hybridization	-	[24]
DNA	2D lattices	base pairing	Buffer/Mg^2+^	[83]
DNA	Tiles	base pairing	Mg^2+^	[84]
DNA	Nanowires/Sheets	base pairing	-	[85]
DNA	2D arrays	base pairing	Ni^2+^	[132]
DNA	Capsules	base pairing	Light	[159]
DNA	Origami	base pairing	Light	[160]
DNA	Origami	base pairing	Light	[161]
RNA	Tetrahedrons	RNA packing	-	[91]
RNA	Triangles	RNA packing	-	[92]
RNA	Lattices/Tubes	RNA packing	-	[93]
**PNA**	Fibers	π–π and base pairing	-	[94]
**Virus**				
CCMV	3D crystals	Ligand-receptor	-	[95]
Bacteriophage P22	P22VLP-NPs	Electrostatic interaction	NPs	[59]
**Enzymes**				
OxOx/HRP	CRGO-enzyme	Hydrophobic	pH	[73]
GOx/CAT	graphene nanodots-porous gold	π-π	Organic	[80]
**Other biopolymers**				
cholesterol	Microrods/ribbons	-	Polymer	[104]
cholesterol	Aggregates	-	Polymer	[105]

CRGO, chemically reduced graphene oxide.
